# Injury Profile SIMulator, a Qualitative Aggregative Modelling Framework to Predict Crop Injury Profile as a Function of Cropping Practices, and the Abiotic and Biotic Environment. I. Conceptual Bases

**DOI:** 10.1371/journal.pone.0073202

**Published:** 2013-09-03

**Authors:** Jean-Noël Aubertot, Marie-Hélène Robin

**Affiliations:** 1 Institut National de la Recherche Agronomique, Unité Mixte de Recherche 1248 Agrosystèmes et Agricultures, Gestion des Ressources, Innovations et Ruralités, Castanet-Tolosan, France; 2 Université Toulouse, Institut National Polytechnique de Toulouse, Unité Mixte de Recherche 1248 Agrosystèmes et Agricultures, Gestion des Ressources, Innovations et Ruralités, Castanet-Tolosan, France; 3 Université de Toulouse, Institut National Polytechnique de Toulouse, Ecole d’Ingénieurs de Purpan, Toulouse, France; University of Florida, United States of America

## Abstract

The limitation of damage caused by pests (plant pathogens, weeds, and animal pests) in any agricultural crop requires integrated management strategies. Although significant efforts have been made to i) develop, and to a lesser extent ii) combine genetic, biological, cultural, physical and chemical control methods in Integrated Pest Management (IPM) strategies (vertical integration), there is a need for tools to help manage Injury Profiles (horizontal integration). Farmers design cropping systems according to their goals, knowledge, cognition and perception of socio-economic and technological drivers as well as their physical, biological, and chemical environment. In return, a given cropping system, in a given production situation will exhibit a unique injury profile, defined as a dynamic vector of the main injuries affecting the crop. This simple description of agroecosystems has been used to develop IPSIM (Injury Profile SIMulator), a modelling framework to predict injury profiles as a function of cropping practices, abiotic and biotic environment. Due to the tremendous complexity of agroecosystems, a simple holistic aggregative approach was chosen instead of attempting to couple detailed models. This paper describes the conceptual bases of IPSIM, an aggregative hierarchical framework and a method to help specify IPSIM for a given crop. A companion paper presents a proof of concept of the proposed approach for a single disease of a major crop (eyespot on wheat). In the future, IPSIM could be used as a tool to help design *ex-ante* IPM strategies at the field scale if coupled with a damage sub-model, and a multicriteria sub-model that assesses the social, environmental, and economic performances of simulated agroecosystems. In addition, IPSIM could also be used to help make diagnoses on commercial fields. It is important to point out that the presented concepts are not crop- or pest-specific and that IPSIM can be used on any crop.

## Introduction

Third millennium agriculture must reconcile environmental protection and productivity. The world population is projected to reach 8.7–10 billion by 2050 and annual_production will need to increase by 200 million tons by then to meet the projected 470 million ton demand [1]. Several authors attribute the spectacular increase of agricultural production in the second half of the twentieth century to the massive use of products resulting from chemical synthesis [2]; but this intensive production model is nowadays questioned because of public health, agronomic, environmental, and sometimes socio-economic issues. Concepts in crop protection in intensive agricultural production systems changed from destruction of pests (by which we mean, plant pathogens and animal pests in this paper) by the use of pesticides to pest management with techniques based on the improved knowledge of pest dynamics and their natural enemies and the interaction between pests and crops under the influence of Cropping Practices [3]. It is therefore necessary to combine cultural, genetic, biological, physical and chemical control methods to manage pests through Integrated Pest Management (IPM) strategies in order to maintain the pest population levels below those causing economic losses [4].

True IPM is quite different from the practices recommended up to now [5] and is still faced with agronomic and technical difficulties which can curb its development. Its impact on pests is difficult to estimate because of their multiplicity and of their many interactions within agroecosystems. Studies on the effects of alternative control methods mostly concern a major pest (monospecific approach) while farmers have to manage an injury profile in a given field, i.e. a combination of injury levels caused by multiple pests (multi-specific approach) [6]. Similarly, the research has focused on the effect of one (or a few) control method(s), but farmers usually combine several operations (which may have only partial effects) to limit pest development. Each technical operation is likely to modify the sanitary status of a crop [7]. In addition, not only do cultural practices interact with each other, but also, one technique can be detrimental to some pests and favourable to others. Pest populations are characterised by a very high level of diversity and complexity because of multiple interactions within and between populations and with biological, physical, and chemical environments. This complexity is one of the constraints to the implementation of IPM, in addition to others [8]. In order to reduce the reliance of cropping systems on pesticides, it is therefore necessary to develop tools to help the “vertical integration” (combination of several control methods) and the “horizontal integration” (simultaneous management of several pests) of IPM strategies. Dynamics of pest populations can lead to combinations of injuries on a crop which can in turn lead to quantitative or qualitative damage, which usually results in economic losses for farmers and more generally for society as a whole. However, these relationships are not linear and depend on the production situation as shown by several authors [6], [9], [10]. In this paper, we will assume that the production situation is defined by the physical, chemical and biological components, except for the crop, of a given field (or agroecosystem) and its environment, as well as socio-economic drivers that affect farmer’s decisions (adapted from [11]). In this definition, the term “environment” refers to the climate and the territory (i.e. landscape and the associated actors) that can directly or indirectly influence the considered field. In a given production situation, a farmer can design several cropping systems according to his goals, his perception of the socio-economic context and his environment, farm organisation, knowledge and his cognition. However, a given cropping system in a given production situation will be assumed to lead to a unique injury profile.

In order to help design cropping systems, modelling is a key tool [12]. However, because of the complexity of agroecosystems, models usually only address a limited part of agroecosystems. Crop models have been developed for decades but do not take into account interactions with pests (e.g. [13], [14]). Epidemiological models *sensu lato* have been developed to represent pest dynamics, often to help decision making for pesticide treatments. However, these models usually take into account rather poorly the critical effects of cropping practices [15] due to their multiple consequences on the crop-pest-environment dynamics [16]. In addition, the majority of these models address single pests (except for models such as EPIPRE [17], [18]). So far, the only models that consider injury profiles are damage models [19], [20]. However these models do not predict injury profiles but the quantitative damage that they cause. There is thus a strong need to develop an innovative approach to predict injury profiles as a function of production situations and cropping practices. Because of the complexity of the considered systems [21], and the lack of representation of the effects of cropping practices and their interactions, the linkage of available crop models to epidemiological models seems unlikely to happen when considering multiple pests [3]. Even if a crop model was available, together with epidemiological models for diseases, weeds and animal pests, taking into account the crop status and the effects of cropping practices, attempting to link them would certainly lead to a dead end because of the propagation error phenomenon as well as the large number of parameters and input variables needed. Alternatively, one could consider statistical approaches to cope with the impossibility of addressing these issues when using mechanistic models. However, datasets with observed injury profiles, cropping systems and production situation are scarce and statistical approaches are thus even more unlikely to succeed than mechanistic modelling approaches. As an alternative, a generic modelling framework, called IPSIM for Injury Profile SIMulator is proposed. It is deliberately simple in the way mechanisms are represented because the system being described, i.e. the agroecosystem, is far too complex for a truly mechanistic representation. It is based on a simple qualitative hierarchical aggregative approach to represent the effects of various factors affecting injury profiles. This paper presents the basic principles of IPSIM, describing its implementation in a software program and providing an example of its specification for a given crop. A companion paper [22] provides a proof of concept of this innovative modelling approach in the field of crop protection for an important disease of wheat.

## Materials and Methods

### Basic Principles of IPSIM

**Figure 1:**
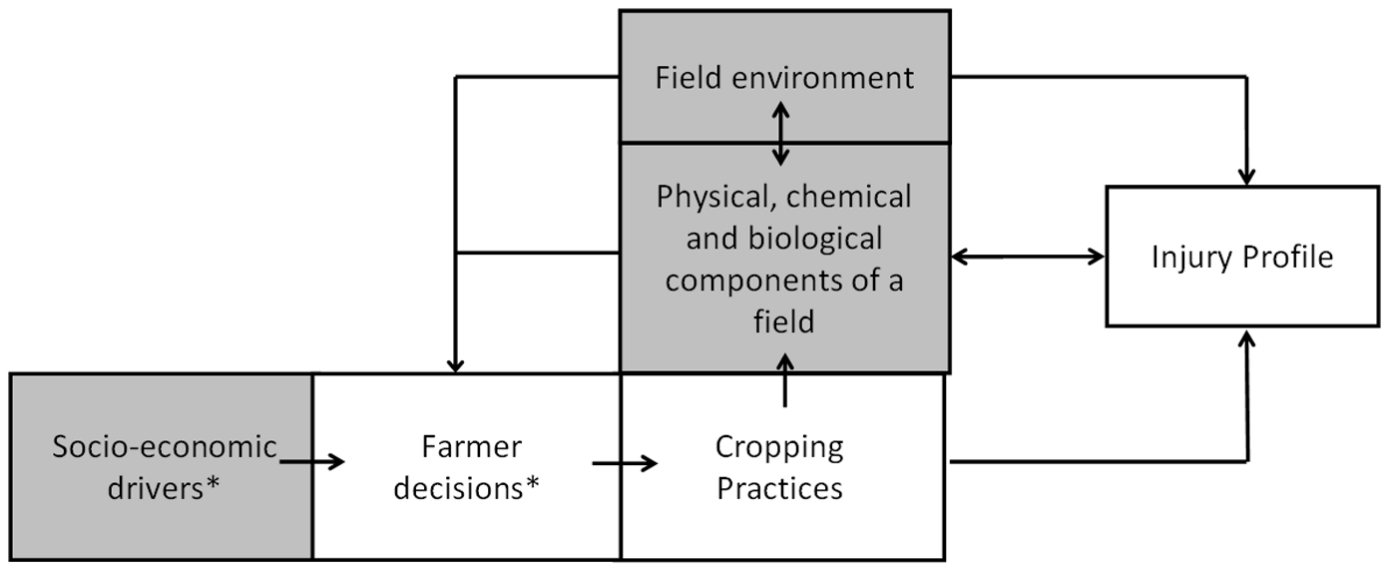
Schematic representation of an agroecosystem and its drivers. In green: components defining the Production Situation (except for the crop). The injury profile is the output variable of IPSIM, whereas its input variables are included within the three following components: cropping practices, field environment, and physical, chemical and biological (crop, pests, beneficials and harmless living organisms) components of the field. *Not taken into account in IPSIM.

Figure 1 is a schematic representation of an agroecosystem. This figure is the conceptual basis of IPSIM, although its scope is broader than the system directly addressed by IPSIM. According to the farmer’s goals, his farm features, his perception of the environment and of the socio-economic context, as well as his knowledge and cognition, he designs cropping systems that will achieve social, economic and environmental performances, as a function of the production situation. These performances will be highly dependent on the injury profile encountered. The term “cropping system” refers here to “a set of management procedures applied to a given, uniformly treated area, which may be a field, part of a field or a group of fields” [23]. This covers many technical operations, for instance, the choice of the crop sequence, cover cropping, cultivar, tillage practices, date and density of sowing, rate of fertilisation and chemical pest control. The term “system” is used here because these technical choices are inter-dependent [24].

IPSIM is embedded in Figure 1, where its output variable is the injury profile. Input variables of IPSIM are embedded within the three following components: cropping practices, field environment, and physical, chemical and biological components of the field (crop, pests, beneficial and harmless living organisms). An injury profile can thus be seen as the result of hierarchical interactions among the cropping practices and the production situation. Qualitative aggregative hierarchical approaches have been used in several fields to help assess the performances of various options when managing a system: industry (e.g. [25], [26]), soil science (e.g. [27]), tourism (e.g. [28], [29]). In the field of agronomy, qualitative aggregative hierarchical models have been used for the assessment of the sustainability of cropping systems *ex-ante* or *ex-post* [30]–[32], the assessment of organic systems [33], the management of Genetically Modified crops (e.g. [34]), the assessment of less-favoured areas for agricultural production (e.g. [35]), the evaluation of energy crops for biogas production (e.g. [36]), the assessment of varieties or cultivars (e.g. [37], [38]) and the assessment of the effects of market-gardening cropping systems on soil borne pathogens and animal pests using expert knowledge of advisors [39]. We used this approach to summarise available knowledge in the literature for a given crop and to develop a generic modelling framework for IPM.

### Implementation of IPSIM with a Software Program

IPSIM was developed using the DEX method, and is implemented with the DEXi software ([40], http://www-ai.ijs.si/MarkoBohanec/dexi.html). DEX is a method for qualitative hierarchical multi-attribute decision modelling and support based on a breakdown of a complex decision problem into smaller and less complex sub-problems. This tool is generally used to evaluate and analyse decision problems [27], [29], [41]. In this study, it is used for the first time to develop a simulation model that represents the behaviour of an agroecosystem and which quality of prediction can be assessed. The modelling framework has the following features [40]. The sub-problems are hierarchically structured into a tree of attributes that represents the “skeleton” of the model. Terminal nodes of the tree, i.e. leaves or basic attributes, represent input variables of the model (and must be specified by the user). The root node represents the main output: an overall assessment of the evaluated scenarios (an injury profile which is defined by cropping practices and elements of the production situation in this case). The internal nodes of the model are called aggregated attributes. All the attributes in the model are qualitative (ordinal and nominal) rather than quantitative (interval) variables. They take only discrete symbolic values usually represented by words. In the DEX method, the aggregation of values up the tree is defined by “utility functions” based on a set of “if-then” aggregation rules. In our approach, we renamed these functions “aggregating tables” since they are not related to the concept of “utility” in decision theory.

### IPSIM Structure

The process of building a DEXi model usually involves the following four steps [40]: (1) identifying the attributes, (2) structuring the attributes, (3) defining attribute scales, and (4) defining the aggregating tables. These steps should be followed for the development of IPSIM using the diagram presented Figure 1. However, only the first three steps can be carried out in a generic way. Only the generic aggregating tables will be described here since most of them are crop-specific.

#### Structure of the attributes used to predict injury profiles

**Figure 2:**
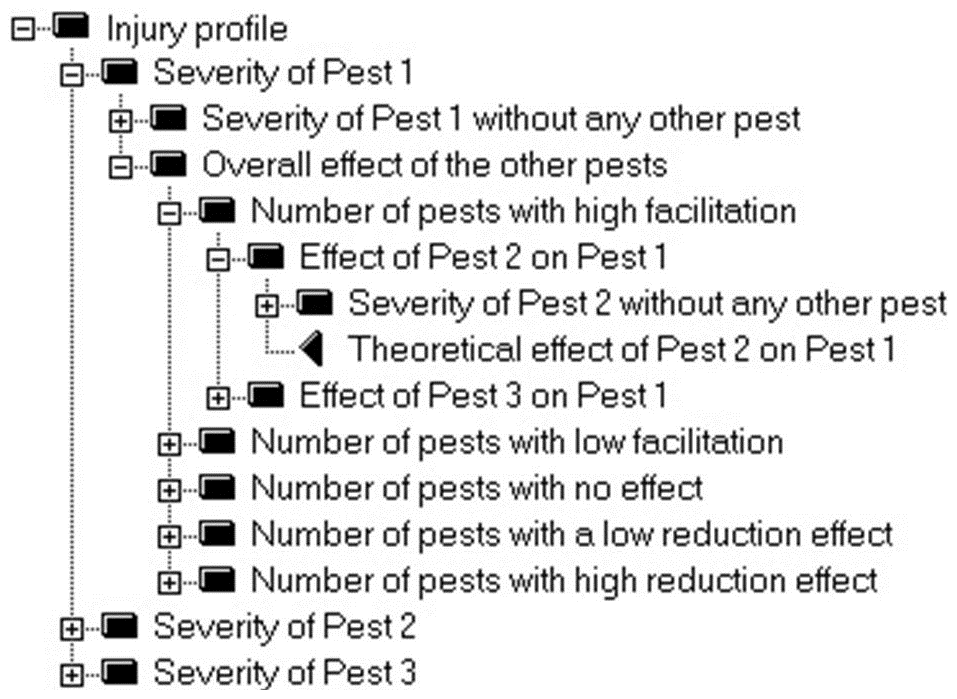
Overall output attributes of IPSIM: description of an injury profile (screenshot of the DEXi software). For the sake of simplicity, only 3 pests are represented in this figure. The severity of a given pest is first calculated independently by IPSIM as if no other pest was present. The aggregated severity of a given pest is then calculated by taking into account the combined effects of all other pests. This is done by considering the theoretical effect of one pest on another according to five levels: high facilitation, low facilitation, no effect, low reduction, high reduction.

The structure of attributes that predict injury profiles is presented in Figure 2. Each injury can take a limited number of severity levels. For instance, 5 classes (very low, low, medium, high, very high) or 7 classes (nil, very low, low, medium, high, very high and maximum) can be considered in IPSIM. Even if only 10 pests and 5 severity levels are considered for a given crop, a theoretical number of 5^10^ = 9.765625×10^6^ possible injury profiles could thus be simulated with IPSIM. This number is only theoretical since some of these injury profiles are impossible due to interactions among pests. In order to take into account these interactions, IPSIM first calculates the severity for single pests independently, as if one pest only was present (Figure 2). Then, interactions between pests are taken into account according the level of each pest and a simple typology of interaction between two pests: high facilitation, low facilitation, no interaction, low reduction, high reduction (Table 1). Table 1 is used to calculate the overall effect of all other pests on the considered pest. Then, the number of pests with high facilitation, low facilitation, no effect, low reduction, high reduction is calculated (Figure 2) and the overall interactions are calculated according to the aggregating tables presented Table 2. Ultimately, the severity of each pest is calculated using the generic aggregating table presented in Table 3 as a function of the severity that would occur without any other pest, and the overall interactions calculated with the aggregating table presented in Table 2.

**Table 1:** Generic aggregating table used to represent the effect of one pest on another in IPSIM.

Severity of Pest 2 without any other pest	Theoretical effect of Pest 2 on Pest 1	Actual effect of Pest 2 on Pest 1
Maximum, very high or high	High facilitation	High facilitation
Maximum, very high or high	Low facilitation	Low facilitation
Maximum, very high or high	No effect	No effect
Maximum, very high or high	Low reduction	Low reduction
Maximum, very high or high	High reduction	High reduction
Medium	High and low facilitation	Low facilitation
Medium	No effect	No effect
Medium	High and low reduction	Low reduction
Low or very low	High facilitation	Low facilitation
Low or very low	Low facilitation, no effect, low reduction	No effect
Low or very low	High reduction	Low reduction
Nil	Any	No effect

**Table 2:** Generic aggregating table used to calculate the overall effect on a given pest caused by all the other pests in an injury profile.

Number of pests withhigh facilitation	Number of pests withlow facilitation	Number of pests withno effect	Number of pests withlow reduction	Number of pests withhigh reduction	Overall effects of allother pests
0	0	0	0	0	No effect
0	0	0	0	≥1	High reduction
0	0	0	≥1	0	Low reduction
0	0	0	≥1	≥1	High reduction
0	0	≥1	0	0	No effect
0	0	≥1	0	≥1	High reduction
0	0	≥1	≥1	0	Low reduction
0	0	≥1	≥1	≥1	High reduction
0	≥1	0	0	0	Low facilitation
0	≥1	0	0	≥1	High reduction
0	≥1	0	≥1	0	Low reduction
0	≥1	0	≥1	≥1	Low reduction
0	≥1	≥1	0	0	Low facilitation
0	≥1	≥1	0	≥1	High reduction
0	≥1	≥1	≥1	0	Low reduction
0	≥1	≥1	≥1	≥1	High reduction
≥1	0	0	0	0	High facilitation
≥1	0	0	0	≥1	Low reduction
≥1	0	0	≥1	0	No effect
≥1	0	0	≥1	≥1	Low reduction
≥1	0	≥1	0	0	High facilitation
≥1	0	≥1	0	≥1	Low reduction
≥1	0	≥1	≥1	0	Low reduction
≥1	0	≥1	≥1	≥1	Low reduction
≥1	≥1	0	0	0	High facilitation
≥1	≥1	0	0	≥1	Low reduction
≥1	≥1	0	≥1	0	Low reduction
≥1	≥1	0	≥1	≥1	High reduction
≥1	≥1	≥1	0	0	High facilitation
≥1	≥1	≥1	0	≥1	Low reduction
≥1	≥1	≥1	≥1	0	No effect
≥1	≥1	≥1	≥1	≥1	Low reduction

**Table 3:** Generic aggregating table used to calculate the severity of one pest in interaction with the other pests of an injury profile.

Severity of the considered pest withoutany other pests	Overall effect of the other pests	Severity of the considered pest under theinfluence of other pests
Maximum	High facilitation	Maximum
Maximum	Low facilitation	Maximum
Maximum	No effect	Maximum
Maximum	Low reduction	Very high
Maximum	High reduction	High
Very high	High facilitation	Maximum
Very high	Low facilitation	Maximum
Very high	No effect	Very high
Very high	Low reduction	High
Very high	High reduction	Medium
High	High facilitation	Maximum
High	Low facilitation	Very high
High	No effect	High
High	Low reduction	Medium
High	High reduction	Low
Medium	High facilitation	Very high
Medium	Low facilitation	High
Medium	No effect	Medium
Medium	Low reduction	Low
Medium	High reduction	Very low
Low	High facilitation	High
Low	Low facilitation	Medium
Low	No effect	Low
Low	Low reduction	Very low
Low	High reduction	Very low
Very low	High facilitation	Medium
Very low	Low facilitation	Low
Very low	No effect	Very low
Very low	Low reduction	Very low
Very low	High reduction	Very low
Nil	High facilitation	Nil
Nil	Low facilitation	Nil
Nil	No effect	Nil
Nil	Low reduction	Nil
Nil	High reduction	Nil

#### Structure of the attributes used to predict the severity of a single pest

**Figure 3:**
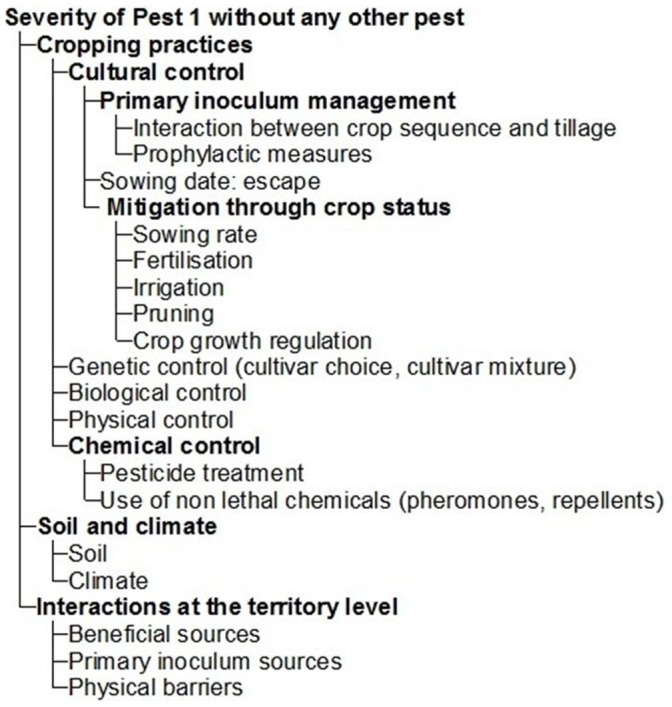
Hierarchical sub-tree to predict the severity of a single pest without any interaction with other pests (screenshot of the DEXi software).

The input attributes of IPSIM describe cropping practices, soil and climate (physical and chemical components of the field which partly define the considered production situation), and biological interactions at the territory level (Figure 1). Figure 3 represents the sub-tree used in IPSIM to calculate the severity of a single pest without any interaction with other pests for a given crop. In this sub-tree, cropping practices are composed of cultural, genetic, biological, physical and chemical control actions. Inocula *sensu lato* are supposed to be non limiting in order to keep basic attributes as simple as possible. The most detailed level is cultural control. It is composed of actions for the management of primary inoculum (through the interaction between crop sequence and tillage for arable crops and prophylactic measures for perennial crops); escape strategies through the choice of the sowing date (some crops are less susceptible to some pests after or before some phenological stages) and mitigation through crop status (as a function of the sowing rate, fertilisation, irrigation, pruning for perennial crops, and application of crop growth regulators). The genetic control represents the level of resistance of the cultivar (or the cultivar mixture) to the considered pest. For some pests, biological control can be applied using living organisms released at the field or greenhouse scale. Physical control consists of using any mechanical, thermal, or electromagnetic actions to limit the pest population. Finally, the attribute “Chemical control” describes the efficacy of pesticide treatments and/or use of non-lethal chemicals such as pheromones or repellents. The effect of soil and climate are described independently and later aggregated in a “Soil and climate” attribute. Finally, the effects of elements (e.g. other fields, hedges, forests) at the territory level are taken into account by describing sources of primary inoculum and beneficials at the territory level, as well as the presence of physical barriers that might limit these interactions between the considered field and its surrounding environment. Harmless living organisms (i.e. neither pests nor beneficials) are not specifically represented in the model.

The scales and the aggregating tables used for the attributes presented in Figure 3 cannot be determined in a generic way. They have to be defined according to experimental results, literature, models, or expert knowledge and are specific to the considered crop and pests.

#### Typology of simulated injury profiles

So far, IPSIM was presented as a simulator of the severity levels for single pests interacting in an injury profile (Figure 2). This detailed information is valuable to researchers, advisers and even farmers to characterise the agronomic performance of cropping practices in a given production situation with regard to potential losses that various pests may cause. However, IPSIM can provide other information, less precise for the injury profile description, but more pertinent for the diagnosis of the overall effects of cropping practices and the biological environment of the considered field on injury dynamics. We chose to categorise pests according to a simple characteristic that describes their level of dependency to the cropping system: their level of endocyclism (high and low). The term “endocyclic” refers to an organism whose development is mostly restricted to a field and highly depends on the field endo-inoculum. The level of endocyclism of a given pest is therefore directly defined by the level of persistence of primary endo-inoculum *sensu lato* in a given field and its dispersal ability. Pests with a high level of persistence and low dispersal ability are highly endocyclic. Pests with a low level of persistence are slightly endocyclic, regardless their dispersal ability. Pests with a high level of persistence and a high dispersal ability are moderately endocyclic. The inoculum produced by an endocyclic pest in one season can be carried over to the next, thus building up a cumulative inoculum reservoir over the years. Endocyclic organisms are thus highly dependent on field history. The categorisation of pests into two groups (high/medium and low levels of endocyclism) can help identify the main level to address to control them: the field or territory level.

For example, root-knot nematodes (*Meloidogyne* spp.) on horticultural crops, wireworms on potato (*Agriotes* spp.), wheat common bunt (*Tilletia* spp.), take-all on wheat (*Gaeumannomyces graminis* var. *tritici*), dicotyledonous weeds such as *Chenopodium album* and *Fallopia convolvulus* are highly endocyclic pests. However, highly endocyclic pests can sometimes be spread to other fields by anthropic activities (e.g. via agricultural machinery, pruning tools, clothes and boots of greenhouse technicians). This dispersal mechanism will not be taken into account in the model. Aphids on several crops (e.g. *Brevicoryne brassicae*), powdery mildew on grapevine (*Erysiphe necator*), rusts on cereals (e.g. *Puccinia recondita*), codling moth on apple tree (*Cydia pomonella*), and weeds such as some *Asteraceae* (e.g. *Taraxacum dens leonis*) or grassy weeds (e.g. *Bromus sterilis*) are slightly endocyclic pests.

**Figure 4:**
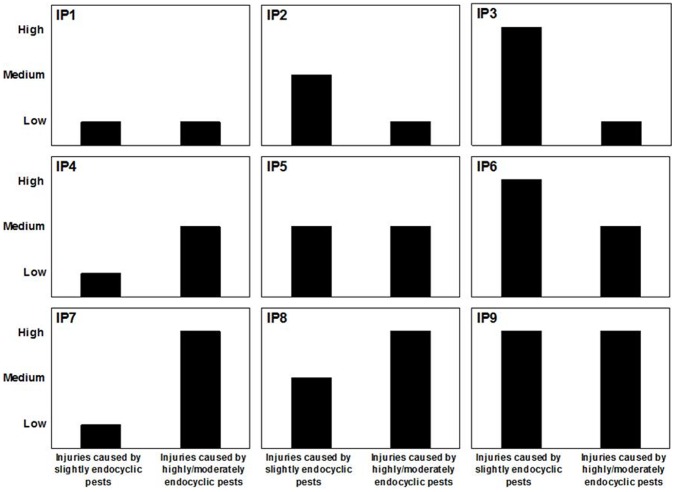
Typology of injuries caused by multiple pests on a crop for given Cropping Practices in a given Production Situation using nine generic Injury Profiles (IP1–IP9). These Injury Profiles are determined by the final levels of the injuries caused by slightly and highly/moderately endocyclic pests (plant pathogens, weeds and animal pests). They can be used to perform cross-cutting analyses for a wide range of agricultural productions.

Two aggregating tables were designed to summarise the distribution of final injury levels of single pests using two aggregated variables: the overall final severity of i) highly/moderately and ii) slightly endocyclic pests (Table 4). Considering three levels of final injury (low, medium, high) for each of the two endocyclism groups, a range of nine possible generic injury profiles was proposed (Figure 4) for any agricultural productions worldwide (i.e. major crops; vegetables; vineyard; orchards; horticulture; industrial crops, aromatic and medicinal plants; grassland; in field or in Controlled Environment Agriculture). For production situations where injury profile haves high final injury levels of highly endocyclic pests (IP7, IP8; IP9; Table 5), a better management of primary inoculum production at the field level should be undertaken (e.g. interaction between by crop sequence and tillage; stubble management, volunteer management, stale seedbeds and sanitation measures for perennial crops). For production situations with injury profiles with high levels of slightly endocyclic pests (IP3, IP6; IP9; Table 5), special attention should be paid to i) the management of inoculum production at the territory level (e.g. spatial distribution of cropping systems, management of primary inoculum production in the neighbouring fields or waste piles, management of interstitial spaces to promote beneficials); ii) escape strategies (sowing date adaptation); iii) mitigation through the crop status (e.g. cultivar choice, sowing rate, nitrogen fertilisation, irrigation).

**Table 4:** Generic aggregating table used to define the level of severity of slightly endocyclic pests in an Injury Profile as a function of the final injury level of single pests.

Number of slightly endocyclic pestswith a very high or maximumfinal injury level	Number of slightly endocyclicpests with a low, medium orhigh final injury level	Number of slightly endocyclicpests with a null or very lowfinal injury level	Overall severity of slightlyendocyclic pests in theInjury Profile
>1	>1	>1	High
>1	>1	0	High
>1	0	>1	High
>1	0	0	High
0	>1	>1	Medium
0	>1	0	Medium
0	0	>1	Low
0	0	0	Low

The same aggregating table is used to define the level of severity of highly/moderately endocyclic pests.

**Table 5:** Equivalence between features of qualitative models developed within the IPSIM framework and quantitative simulation models.

Feature	Qualitative simulation models such as theones developed with the IPSIM framework	Quantitative simulation models
Type of input variables	Nominal, ordinal, or interval	Interval
Type of state variables	Ordinal	Interval
Type of output variables	Ordinal (can be transformed into static intervalor even dynamic interval)	Interval
Model structure	Aggregation tree	Equations
Specification of the model structure	Aggregating tables	Parameters
Analysis of model’s behaviour	Table of local and global weights for eachinput and aggregated attributes	Sensitivity analyses to inputvariables and parameters
Measures of agreement (non exhaustive)	Proportion of correctly predicted ordinal classes;non parametric Wilcoxon signed rank test to analyseif the distribution of errors is significantly biasedor not; matched marginal distribution analysisor joint distribution analysis in a squarecontingency table	Bias; Mean Absolute Error; Root MeanSquared Error; Efficiency

## Results

### Implementation of IPSIM Generic Framework into a Simulation Model, an Example

This article aims to present the whole modelling process: i) development of a conceptual framework; ii) implementation of this conceptual scheme into a simulation model for a simple case; iii) simulation to exemplify potential uses of IPSIM models. The specification of IPSIM will be performed for a simple injury profile on wheat: two highly endocyclic diseases (eyespot and sharp eyespot) and a slightly endocyclic disease (brown rust). Eyespot, caused by the necrotrophic and soil-borne fungi *Oculimacula yallundae* and *O. acuformis*, anamorph *Pseudocercosporella herpotrichoides* is considered to be the most important stem-base disease of cereals in temperate countries. In France, sharp eyespot, another soil-borne fungus caused *by Rhizoctonia cerealis,* is one of the minor diseases of the foot disease complex of winter wheat, but is thought to interact strongly with eyespot. The two pathogens show distinct antagonistic behaviour within the infected stem base, which translates into a negative correlation between sharp eyespot and eyespot incidence [42]–[44]. Finally, brown rust, caused by *Puccinia triticina*, is the most common rust disease of wheat and is now recognised as an important pathogen in wheat production worldwide, causing significant yield losses over large geographical areas [45]. As opposed to the first two soil-borne diseases which are disseminated over short distances, brown rust is an obligate, airborne disease with conidia which are wind-dispersed over hundreds of kilometres, resulting in rust epidemics on a continental scale [46].

The design of IPSIM-Wheat-Eyespot and the evaluation of its predictive quality is described in a companion paper [22]. For the sake of simplicity and readability, the other two models will not be presented in detail, but their development was similar to the one presented in [22]. The two models for eyespot and sharp eyespot are similar in terms of structure (tree) and aggregating tables because the impact of cropping practices on sharp eyespot is similar to that on eyespot [42]. However, since brown rust is an airborne disease, the effects of primary inoculum management at the field scale are less important than for the two soil-borne diseases. For this airborne disease, the main control methods are: i) mitigation through crop status (using a resistant cultivar for instance) and; ii) the management of primary inoculum sources at the territory level. For this schematic injury profile, we will assume that no direct interactions occur between the two soil-borne, stem-base diseases and this airborne, foliar disease.

### Simulation Scenarios

**Figure 5:**
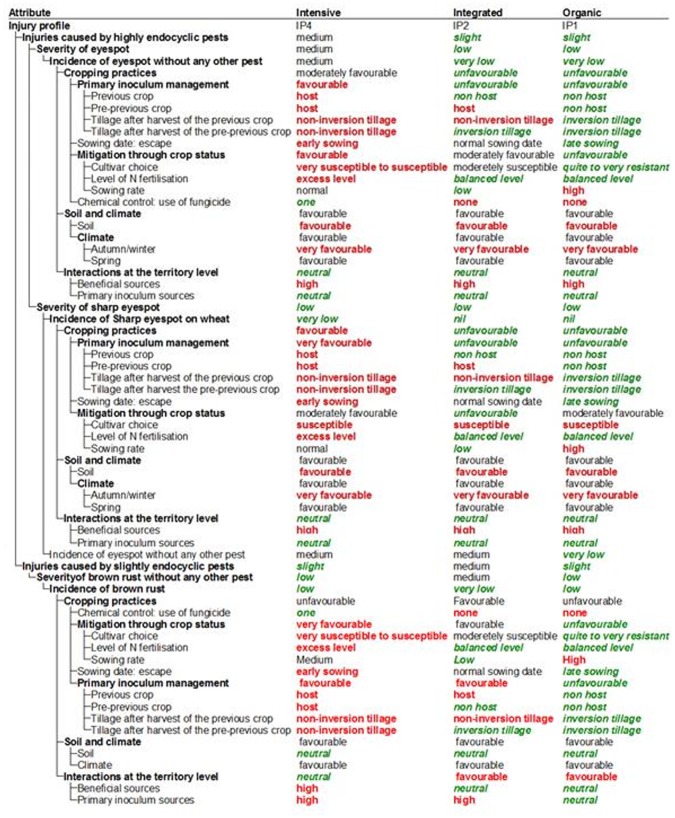
Example of simulation outputs for wheat obtained for three cropping systems (intensive, integrated and organic) in a given production situation (screenshot of the DEXi software). Three pests in interaction were taken into account in these simulations: eyespot, sharp eyespot and brown rust.

The use of the model presented in the first sub-section of the “results” section is exemplified for three contrasting cropping practices in a given production situation (Figure 5). The three cropping practices considered were: intensive, integrated and organic systems. The intensive system is a wheat monoculture with a high level of inputs and a high-yielding cultivar susceptible to diseases, aiming at a high yield level. The integrated system is characterised by a limited use of inputs, with a lower-yielding cultivar than the former system, but less susceptible to diseases, a short wheat rotation, and a satisfactory yield level. The organic system is characterised by low inputs, with a disease-resistant cultivar with a limited yield, associated with a long wheat rotation and appropriate crop management. The three systems were tested in the same production situation, with a weather scenario favourable to the development of the considered diseases.

### Simulation Results

The DEXi software computed the aggregated attribute values of the model presented in the first sub-section of the “results”. In the same production situation, the three cropping practices led to contrasting injury profiles. In the absence of estimates of potential yield losses caused by these injury profiles, it is difficult to provide direct recommendations for cropping practices adaptations. However, these simulations enable a diagnosis in terms of pest development for the three simulated systems. The intensive system led to IP4, i.e. a medium final injury level for highly endocyclic pests associated with a low final injury level for slightly endocyclic pests (Figure 5). For this system, the model suggests that a better management of primary inoculum of the pathogen responsible for eyespot injury should be considered. The integrated system led to IP2, i.e. a low final injury level for highly endocyclic pests associated with a medium final injury level for slightly endocyclic pests (Figure 5). For this system, the model suggests that a better control of brown rust through the use of a more resistant cultivar or the use of a low-dose fungicide, provided that it would be economically sound. The organic system led to IP1, i.e. a low final injury level for highly endocyclic pests associated with a low final injury level for slightly endocyclic pests (Figure 5). This is consistent with the associated cropping practices which aims at minimising pest development by combining prophylactic measures with partial effects. It is important to underline that this diagnosis did not address yield losses, but focused only on injury.

## Discussion

### Potential Uses of IPSIM Models

These simulations illustrate how IPSIM can be used to assess *ex-ante* the performance of various cropping systems with regard to the control of pest injury on a given crop. This information is useful when designing innovative cropping systems, either by prototyping, e.g. [47], simulation, e.g. [48], or expert knowledge, e.g. [12]. Since climate significantly affects injury profiles, weather frequency analyses are needed, using a set of input variables describing a wide range of climatic scenarios so that the information provided by IPSIM is robust in the face of weather variability. However, IPSIM cannot be seen as a model to design innovative cropping systems *in silico* for two major reasons. First, crop damage is not simulated by IPSIM, which makes it difficult to rank pests with respect to the crop losses they cause. Second, the social, economic and environmental performance of the simulated cropping systems are not calculated. To tackle this problem, IPSIM could be coupled to a damage model (such as RICEPEST [6], [19], [20] or WHEATPEST [20]) that would predict yield losses as a function of the injury profiles encountered and other relevant variables. Alternatively, a crop model (e.g. STICS [14] ) could be used, with a set of single damage functions (such as the ones used in WHEATPEST [20]), and coupled with IPSIM. Then, once the damage caused by a given injury profile in a given production situation has been predicted, a more general framework, such as MASC, [30] or DEXiPM, [49], could be used to predict the social, economic and environmental performance of the tested systems in a given production situation. This approach will help design innovative cropping systems less vulnerable to pests. Using that modelling framework, IPSIM would be the missing link to fill the gap between crop models that can help predict performance of pest-free cropping systems and epidemiological models that generally do not represent the effects of crop status under the influence of cropping practices. In addition, models developed with IPSIM could be used to create typologies of injury profiles at a regional, national, continental or even worldwide scale, using a schematic description of soil and climate, together with a description of the diversity of cropping practices. This should reveal the main injury profiles encountered and help design strategies to control them with better vertical and horizontal integration of IPM. If the corresponding damage models were available, the typology produced could help prioritise objectively research efforts on the main harmful pests.

IPSIM could also be used in an *ex-post* analysis to understand the behaviour of commercial field or experimental plots. Finally, it can be viewed as a communication tool for groups, as well as to teach practitioners and students. Knowledge of several scientific fields involved in crop protection, as well as several types of expertise (of scientists, extension engineers, or farmers) can be built into IPSIM, offering a framework for these various communities to interact and combine their knowledge.

### Limitations of the Approach

Like any other model, the predictive quality of IPSIM should be assessed prior to its use. This highlights the urgent need to collect data in commercial fields describing the input and output variables of IPSIM (*i.e.* cropping practices, soil and climate, field environment, injury profiles), along with the social, economic and environmental performances of the monitored agroecosystems. It is important to also add measurements of state variables characterising the crop status (e.g. biomass per area unit, Leaf Area Index) in order to better describe important state variables of the agroecosystem for other possible future analyses of the created datasets. However, due to the lack of datasets containing a description of injury profiles, the confidence that users may have in IPSIM models could also be enhanced by comparing simulation outputs with their own expertise to identify any mismatches. All the information contained within IPSIM models is held in the hierarchical trees and the associated aggregating tables. One of the consequences of this specificity of models developed with the IPSIM framework is that, once developed, the predictive quality of the models can be enhanced easily using experimental datasets by modifying aggregating tables, and, if need be, the structure of the model.

The possible injury profiles that IPSIM models can simulate are numerous. However, observations tend to show that the diversity of injury profiles encountered in commercial fields is much less than the structure of IPSIM models can generate. This results from two mechanisms. First, pests can interact directly (through facilitation, predation, competition for the same ecological niche) or indirectly (though modification of the biotope). This implies that not all potential theoretical injury levels could occur simultaneously. This is a limitation of IPSIM which does not account for the impact of injuries on crop growth. Secondly, the soil, climate, cropping practices and landscape occurring in a given territory might not be diverse enough to lead to all theoretical injury levels (for instance, the theoretical injury profile with all the forms of injury at their maximum level does not exist in reality). Another limitation of IPSIM is the way that interactions between pests are represented. If n pests are considered, n (n-1) interactions are to be described. This is similar to the three-body (or n-body) problem in physics, which has a global analytical solution in the form of convergent power series [50], but that has to be approximated in practice because they converge too slowly. IPSIM models approximate interactions among injuries by arbitrarily calculating the global interaction that would occur between a given injury and the rest of the injury profile defined as the sum of single injuries simulated without taking into account interactions among pests. However, this approximation certainly appears negligible as compared to other necessary simplification hypotheses.

From the conceptual viewpoint, it could be asked why the crop which is entered in the field biological component (Figure 1) does not appear at the first level of the IPSIM tree. After all, pests only experience physical, chemical and biological interactions within agroecosystems and a description of i) the crop status, ii) soil and climate, and iii) the neighbouring environment of the field are indeed the true drivers of pest dynamics. This option was tried when developing IPSIM structure, but led to too complicated a structure, the effect of single cultural operations being overlooked among the numerous levels of the tree. In addition, datasets with a description of cropping practices and injury profiles are extremely scarce. The requirement of additional variables describing the crop status (e.g. in terms of phenology, architecture, biomass, Leaf Area Index) would also lead to greater difficulties in developing IPSIM models and in evaluating its predictive quality.

We recommend to develop models with no more than 7 final injury levels for a single pest. The lack of precision of IPSIM models could be seen as a drawback as compared to quantitative epidemiological models. Firstly this is because these latter models address a much simpler system: a single pest, rather than an injury profile. Secondly, when developing models of complex systems, accuracy should be sought rather than precision. Searching for better precision would certainly lead to an increase in the model’s complexity and possibly to a dead end. We believe that the proposed precision of the models that will be developed with the IPSIM framework is more than enough for the main ultimate purpose of the model: helping the design of innovative cropping systems less vulnerable to pests.

### Points for Reflection

The presented structure of IPSIM is not exhaustive in terms of control methods that can be undertaken. However, developers of models within the IPSIM framework can always easily modify its structure in order to take into account the effects of control measures not present in Figure 3. For instance, the effect of cultivar mixtures or intercrops could be implemented, provided that the required knowledge is available.

The main breakthrough of IPSIM is to be able to handle complexity in a simple way. Input variables of the IPSIM models should be simple to provide. Most of these input variables will be static variables, except for weather variables that will be dynamic. The price to pay to handle the level of ecological complexity (such as defined by Li [51]) addressed by IPSIM is that IPSIM models are static. This is certainly not a problem to predict the consequences of technical options in a given production situation, but could hamper the linkage with dynamic models as suggested earlier. This limitation could easily be overcome by associating the level of final injury predicted by IPSIM models with generic dynamics. In order to do so, exponential, monomolecular, logistic, Gompertz, or Richards models [52] could be used with generic parameters chosen to represent the qualitative ordinal different injury levels predicted by IPSIM models.

The choice of qualitative variables to describe agroecosystems is relevant for several reasons. Firstly, farmers generally rely on a qualitative perception of their environment to make decisions. This suits the formalism of IPSIM. Secondly, because of the complexity of the system, few datasets are available to describe its components, i.e. the production situation, cropping practices and the injury profile. Using qualitative variables enables one to gather and use various existing datasets that were not acquired for the development of IPSIM models. For instance, datasets from diagnoses of commercial fields or even from experiments may not have used the same severity scale for a given disease. The use of qualitative classes allows data from different origins and/or with different precision to be combined. It is possible to associate interval classes with qualitative attributes. For instance, the 7 levels “nil”; “very low”; “low”; “medium”; “high”; “very high”; and “maximum” can be transformed into [0]; [0–20]; [20]–[40]; [40–60]; [60–80]; [80–100]; [100] intervals of percentage of diseased foliage respectively, if one wants to compare these outputs with observed severities of a disease for instance. Thus, data acquired on various scales can still be combined to strengthen the dataset used to estimate the predictive quality of the model or to improve the aggregating tables.

Table 5 presents the equivalence between features of models developed within the IPSIM framework and more common quantitative simulation models. Input attributes of IPSIM models can be nominal, ordinal or interval variables, unlike quantitative simulation models, which require only interval input variables. The state variables (aggregated attributes) of IPSIM models, including output variables, are ordinal. However, if need be, output variables of IPSIM models can be transformed into interval variables. This transformation can be performed by associating each possible ordinal value with a quantitative value (e.g. static final value of an injury level, or quantitative intervals) or with an injury dynamic. The relationship between variables is described by a tree in aggregative qualitative models, whereas quantitative models use equations. The DEXi software [40] provides a table with the respective weights of input and aggregated attributes on the value of the root node (main output). This table can be seen as an equivalent to a simple sensitivity analysis to input variables for quantitative models, prior to more detailed ones [53]. It is notable that IPSIM models have no parameters. The equivalents of parameters that specify relationships among variables in quantitative models are the aggregating tables. The proportion of situations correctly simulated is a criterion that can be used to characterise the agreement between values simulated with an IPSIM model and observations. In addition, a non-parametric Wilcoxon signed rank test can be used to analyse whether the distribution of errors is significantly biased or not. These criteria can be seen as equivalent to common statistical criteria for quantitative models (Bias, Mean Absolute Error; Root Mean Square Error; Efficiency [54]). At last, methods specific to matched-pairs data with ordered categories can be used. In order to do so, various models comparing matched marginal distributions or analysing the joint distribution in a square contingency table can be applied [55].

The qualitative attributes of IPSIM models can lead to threshold effects. In order to cope with this limitation, a tool, named proDEX was developed to model uncertain expert knowledge [56]. This software offers the definition of probabilistic aggregating tables, where each combination of descendants’ values maps to a probability distribution of the aggregated attributes, rather than a single value. In this approach, input values must be categorised prior to their use in the model. Since this process is time-consuming, proDEX allows categorisations to be part of the model definition and the inputs to be entered as interval variables. In combination with probabilistic aggregating tables, categorisations can be made to transform numerical values into probabilistic distributions, eliminating the problem of crisp interval boundaries. Eventually, the proDEX method could permit a useful extension of the modelling approach presented in this paper.

Finally, a website giving online access to all the functionalities of IPSIM is planned. This website will enable researchers, advisors, farmers and students to develop their own models for a wide range of crops.

## Conclusion

We believe that IPSIM is a useful innovative modelling framework to help vertical and horizontal integrations for IPM. Its output attributes include nine generic injury profiles that are based on a two-level categorisation of the degree of endocyclism of harmful organisms. These nine injury profiles can be seen as a tool to perform cross-cutting typologies of agroecosytems for various types of crop (arable crops, vegetables, orchards, vineyards, Controlled Environment Agriculture), with regard to the main pests that have to be managed. IPSIM will generate new knowledge by combining various sources of information from experiments, diagnoses of commercial field, models, and expert panels in a simple way, despite the high ecological complexity of the system addressed. The associated companion paper provides a proof of concept of the proposed method for a single pest.
